# Mice Long-Term High-Fat Diet Feeding Recapitulates Human Cardiovascular Alterations: An Animal Model to Study the Early Phases of Diabetic Cardiomyopathy

**DOI:** 10.1371/journal.pone.0060931

**Published:** 2013-04-11

**Authors:** Sebastián D. Calligaris, Manuel Lecanda, Felipe Solis, Marcelo Ezquer, Jaime Gutiérrez, Enrique Brandan, Andrea Leiva, Luis Sobrevia, Paulette Conget

**Affiliations:** 1 Instituto de Ciencias, Facultad de Medicina Clínica Alemana Universidad del Desarrollo, Santiago, Chile; 2 Centro de Regulación Celular y Patológica, Departamento de Biología Celular y Molecular, MIFAB, Pontificia Universidad Católica de Chile, Santiago, Chile; 3 Cellular and Molecular Physiology Laboratory (CMPL), Division of Obstetrics and Gynecology, School of Medicine, Faculty of Medicine, Pontificia Universidad Católica de Chile, Santiago, Chile; 4 The University of Queensland Centre for Clinical Research, Herston, Queensland, Australia; I2MC INSERM UMR U1048, France

## Abstract

**Background/Aim:**

Hypercaloric diet ingestion and sedentary lifestyle result in obesity. Metabolic syndrome is a cluster of clinical features secondary to obesity, considered as a pre-diabetic condition and recognized as an independent risk factor for cardiovascular diseases. To better understand the relationship between obesity, metabolic syndrome and cardiovascular disease as well as for the development of novel therapeutic strategies, animal models that reproduce the etiology, course and outcomes of these pathologies are required. The aim of this work was to characterize the long-term effects of high-fat diet-induced obesity on the mice cardiovascular system, in order to make available a new animal model for diabetic cardiomyopathy.

**Methods/Results:**

Male C57BL/6 mice were fed with a standardized high-fat diet (obese) or regular diet (normal) for 16 months. Metabolic syndrome was evaluated testing plasma glucose, triglycerides, cholesterol, insulin, and glucose tolerance. Arterial pressure was measured using a sphygmomanometer (non invasive method) and by hemodynamic parameters (invasive method). Cardiac anatomy was described based on echocardiography and histological studies. Cardiac function was assessed by cardiac catheterization under a stress test. Cardiac remodelling and metabolic biomarkers were assessed by RT-qPCR and immunoblotting. As of month eight, the obese mice were overweight, hyperglycaemic, insulin resistant, hyperinsulinemic and hypercholesterolemic. At month 16, they also presented normal arterial pressure but altered vascular reactivity (vasoconstriction), and cardiac contractility reserve reduction, heart mass increase, cardiomyocyte hypertrophy, cardiac fibrosis, and heart metabolic compensations. By contrast, the normal mice remained healthy throughout the study.

**Conclusions:**

Mice fed with a high-fat diet for prolonged time recapitulates the etiology, course and outcomes of the early phases of human diabetic cardiomyopathy.

## Introduction

Obesity is a major global health issue. Change in lifestyle, predominantly hypercaloric diet ingestion and sedentary habits result in a dramatic increase in its incidence. Most obese patients develop metabolic syndrome, a cluster of clinical features characterized by insulin resistance and dyslipidaemia [1], [2]. This pre-diabetic condition has been recognized as an independent risk factor for cardiovascular diseases, including: hypertension, atherosclerosis and diabetic cardiomyopathy [3], [4]. In order to better understand the relationship between obesity, diabetes and cardiovascular diseases as well as for the development of novel therapeutic strategies, animal models that reproduce the etiology, course and outcomes of these conditions are required. Genetically-modified and diet-induced mice are currently available [5]. While the former progresses quickly and exhibit an overstated phenotype, the latter shares the etiology and progressive evolution of human diseases. Hence the possibility of extrapolating the data from diet-induced animal models is expected to be greater.

To reproduce cardiovascular human diseases in animal models diverse diet composition have been tested. It has been shown that a high-sucrose diet exacerbates insulin resistance [6], a high-salt or high-fructose diet exacerbates hypertension [7], [8], a high-cholesterol diet exacerbates atherosclerosis [9], and a short-term high-fat diet results in discrete systolic dysfunction [10]. Nevertheless, none of these strategies has been described as an animal model for diabetic cardiomyopathy. Indeed, the Animal Models of Diabetic Complications Consortium currently encourages scientists to propose new animal models of diabetic cardiovascular complications [11]. The availability of a validated and comprehensively characterized animal model of diabetic cardiomyopathy will be useful not only to better understand the disease but also to test new therapeutic strategies [12].

The aim of this study was to characterize the long-term effects of high-fat diet-induced obesity on the cardiovascular system of mice, in order to establish an animal model for diabetic cardiomyopathy. C57BL/6 mice were chosen because they are genetically susceptible to develop insulin resistance [13]. The diet used was elected because it is standardized, commercially available, supplies levels of fat higher than those recommended in a human healthy diet (60 vs. 25% calories from fat, respectively) [14], and proved to induce obesity and non-alcoholic steatohepatitis in C57BL/6 mice. [15], [16]. After 8, 12 and 16 months under this regimen (obese), metabolic, vascular and cardiac features were assessed, and compared with sex- and age-matched mice fed with regular diet (normal).

## Materials and Methods

### Animals

C57BL/6 male mice were housed at constant temperature (22±2°C) and humidity (60%), with a 12∶12 hours light:dark cycle and unrestricted access to food and water. When required, animals were lightly anesthetized with sevofluorane (Abbott Laboratories, Illinois, USA) or 60 mg/Kg ketamine plus 4 mg/Kg xylazine. When sacrificed, animals were deeply anesthetized and received an overdose of ketamine/xylazine (60/4 mg/Kg). Animal protocols were approved by the Ethics Committee of the Faculty of Medicine at Clínica Alemana-Universidad del Desarrollo.

### Obesity Induction

All mice were fed with a regular diet up to until five weeks of age. Then, they were kept on a regular diet (normal) or switched to high-fat diet (obese) up to the end of the study (16 months of tested diet). Regular diet corresponded to 10 cal% fat, 20 cal% proteins and 70 cal% carbohydrates (Champion SA, Santiago, Chile). High-fat diet corresponded to 60 cal% fat, 20 cal% proteins and 20 cal% carbohydrates (D12492, Research Diets Inc., NJ, USA).

#### Blood glucose, insulin, triglyceride and cholesterol quantification

After four hours of fasting, blood samples were collected from the tail vein of alert mice. Plasma glucose levels were determined with the glucometer system Accu-Chek Performa (Roche Diagnostic, Germany). Plasma insulin levels were assayed using ultrasensitive mouse insulin ELISA kit (Mercodia, Uppsala, Sweden). Plasma triglyceride and cholesterol levels were determined using TG Color GPO/PAP and Colestat kits (Wiener Lab, Rosario, Argentina), respectively.

### Glucose Tolerance Test

After four hours of fasting, mice were lightly anesthetized and received intraperitoneally 2 mg D-glucose/g body weight. Fifteen minutes before and 15, 30, 60, 90 and 120 minutes after D-glucose administration, blood glucose quantification was performed. Area under the curve (AUC) was calculated according to the trapezoidal rule as described [13].

### Systolic Blood Pressure Measurement

Systolic blood pressure (SBP) was measured using a sphygmomanometer LE5001 (Panlab, Barcelona, Spain) in conscious mice as previously described [17]. Measurements were taken on three different days using three different settings, averaging at least six readings.

### Vascular Reactivity

Mice were euthanized and thoracic aorta was excised and placed in Petri dishes containing Krebs buffer solution (KBS (mM): 118.5 NaCl, 4.7 KCl, 2.5 CaCl_2_, 1.2 MgSO_4_7H_2_O, 1.2 KH_2_PO_4_, 25 NaHCO_3_, 5.5 D-glucose (pH 7.4, 37°C)). After cleaning loose connective tissue, 2-mm aortic ring segments were mounted on a 610 M Multiwire Myograph (Danish Myo Technology A/S, Denmark). The myograph chambers were filled with KBS at 37°C and constantly bubbled with a mixture of 95% O_2_/5% CO_2_ (pH 7.4). Optimal internal diameter was adjusted from maximal active response to 62.5 mM KCl. The concentration-dependent response to acetylcholine (0.1 nM –1 mM), sodium nitroprusside (1 nM –0.1 mM), and norepinephrine (0.1 nM –0.1 mM) was determined in 32.5 mM KCl-preconstricted aortic rings as described [18]. Mechanical activity was recorded isometrically using a force transducer coupled to a Powerlab 4/30 data acquisition system (AdInstruments, Bella Vista, Australia) with LabChart 7Pro software (ADInstruments, Bella Vista, Australia). Tissue responses were expressed as a percentage of maximal contraction induced by 32.5 mM KCl [19].

### Cardiovascular Parameter Assessment at Basal and Stress Conditions

Mice were deeply anesthetized and placed in supine position on a thermo-regulated plate. Body temperature was monitored using a rectal thermometer and gaseous oxygen was supplied. Hemodynamic parameters were measured by cardiac catheterization [20], [21]. The catheter used consisted of a Mikro-Tip SPR-671 pressure sensor (Millar, Houston, USA), which was coupled to the PCU-2000 transducer pressure/volt (Millar) and connected to the PowerLab 4/30 data acquisition system (AdInstruments, Bella Vista, Australia). Hemodynamic parameters were recorded at basal and stress conditions. For the latter, a PE-10 plastic tube (Warner Instruments Co, CT, USA) was introduced into the mice jugular vein and connected to a KDS-KDS210P pump (Kdscientific Inc., MA, USA) for dobutamine stimulation. Dobutamine is a β-adrenergic agonist with a high affinity for β_1_-receptors expressed in the heart. When systemically administered, it increases cardiac demand producing cardiac stress. The dobutamine infusion regime consisted in six, two-minute intervals, from 2 ng/g/min to 12 ng/g/min [22]. Data obtained were analysed with LabChart 7Pro software (AdInstruments, Bella Vista, Australia).

### Transthoracic Echocardiographic Assessment

Mice were deeply anesthetized and placed in supine position on a thermo-regulated plate at 37°C. Images in parasternal position at the level of the papillary muscles were obtained with Logiq Book XP enhanced ultrasound equipment using a linear transducer of 10 MHz (i12L-RS, GE Healthcare, USA). The electrocardiographic signal was obtained with electrodes (USB-ECG, GE Healthcare, USA). The thickness of the ventricular walls and the left ventricular internal diameter at diastole (LVId) and systole (LVIs) were determined from images in mode B and M. The shortening fraction (FS) was calculated according to the formula as described [23]

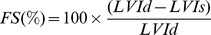



### Cardiac Macroscopic Analysis

Mice were euthanized and heart and tibia were dissected. The heart was washed in 0.9% NaCl and weighed. The tibia was heated in 0.1 M NaOH for 10 minutes and its length was measured with a calliper. Heart weight was normalized against tibia length [24].

### Cardiac Histologic Analysis

Mice were euthanized and heart was dissected, washed in 0.9% NaCl and fixed in 10% paraformaldehyde for 48 hours. Next, it was dehydrated with ethanol and mounted in paraffin. Sections of five µm thickness were stained with haematoxylin-eosin (H&E) or with biotinylated-IsoLectin B4, streptavidin conjugated with HRP and peroxidase ImmPACT DAB substrate kit (Vector, CA, USA). An assessment of cardiomyocyte cross-sectional area (A[cmy]) and the number of capillaries per cardiomyocyte were performed as described [25].

### Cardiac Protein Analysis

Samples of twenty-five mg of heart were homogenized in 500 µl of 50 mM Tris–HCl, pH 7.4, 0.1 M NaCl, 0.5% Triton X-100, 1% SDS with 1 mM phenylmethylsulfonyl fluoride. Aliquots were subjected to SDS gel electrophoresis in 8% polyacrylamide SDS-PAGE gel, transferred onto nitrocellulose membrane (Schleicher and Schuell, Keene, USA), probed with antibodies against collagen type I (Abcam, MA, USA), type III (Rockland, PA, USA) or tubulin (Sigma, USA), and revealed using an enhanced chemiluminescence kit (Pierce, IL, USA). Densitometric analysis and protein semi-quantification were performed using ImageJ software [26].

#### Cardiac gene expression analysis

Total RNA was purified using TRIzol (Invitrogen, CA, USA). One µg of total RNA was used for reverse transcription. Real-time PCR reactions were performed in a final volume of 10 µl containing 50 ng of cDNA, PCR LightCycler-DNA Master SYBRGreen reaction mix (Roche, IN, USA), 3 mM MgCl_2_ and 0.5 µM of each primer (Table 1), using a LightCycler 1.5 thermocycler (Roche). To ensure that amplicons were from mRNA and not from genomic DNA amplification, controls without reverse transcription were included. Amplicons were characterized according to their size and melting temperature (Tm). The mRNA level of a target gene was standardized against the mRNA level of *GAPDH*, from the same sample. Results are presented as fold of changes *versus* normal mice [27].

**Table 1:** Characteristics of Primers and Amplicons.

Gene	Primers		Amplicons		
Names	Forward sequences (5′ to 3′)	Reverse sequences (5′ to 3′)	Tm (°C)	Size (bp)	
***GLUT1***	GCTGTGCTTATGGGCTTCTC	CACATACATGGGCACAAAGC	57	114	
***GLUT4***	ACATACCTGACAGGGCAAGG	CGCCCTTAGTTGGTCAGAAG	59	152	
***PPAR-α***	AGAGCCCCATCTGTCCTCTC	ACTGGTAGTCTGCAAAACCAAA	61	153	
***PDK4***	GCATTTCTACTCGGATGCTCATG	CCAATGTGGCTTGGGTTTCC	59	79	
***UCP3***	CTGCACCGCCAGATGAGTTT	ATCATGGCTTGAAATCGGACC	59	191	
***α-MHC***	GCCCAGTACCTCCGAAAGTC	GCCTTAACATACTCCTCCTTGTC	58	110	
***β-MHC***	AGGGCGACCTCAACGAGAT	CAGCAGACTCTGGAGGCTCTT	60	114	
***SERCA2A***	TGAGACGCTCAAGTTTGTGG	ATGCAGAGGGCTGGTAGATG	58	145	
***COL I***	AGAACATCACCTATCACTGCAAGA	GTGGTTTTGTATTCGATGACTGTCT	61	205	
***COL III***	TCGGAACTGCAGAGACCTAAA	CCCCAGTTTCCATGTTACAGA	56	122	
***GAPDH***	ACTCCACTCACGGCAAATTC	TCTCCATGGTGGTGAAGACA	58	171	

*COL I* : collagen type I, *COL III*: collagen type III, *α-MHC*: alpha-myosin heavy chain, *β-MHC*: beta-myosin heavy chain, *SERCA2A*: sarco/endoplasmatic reticulum calcium ATPase, *GLUT1:* glucose transporter 1, *GLUT4:* glucose transporter 4, *PPAR-α*: peroxisome proliferator-activated alpha receptor, *PDK4:* pyruvate dehydrogenase kinase isozyme 4, *UCP3:* uncoupling protein 3, *GAPDH*: glyceraldehyde-3-phosphate ehydrogenase.

#### Statistical analysis

Data are presented as mean ± S.E.M. To determine the statistical significances of intergroup differences, two-way ANOVA test was used to compare mean values among all groups and Student’s unpaired *t*-test or Mann-Whitney test (non parametric) was used to compare mean values between two groups. p<0.05 was considered as statistically significant.

## Results

### High-fat Diet Induces Obesity and Metabolic Syndrome in Mice

**Figure 1:**
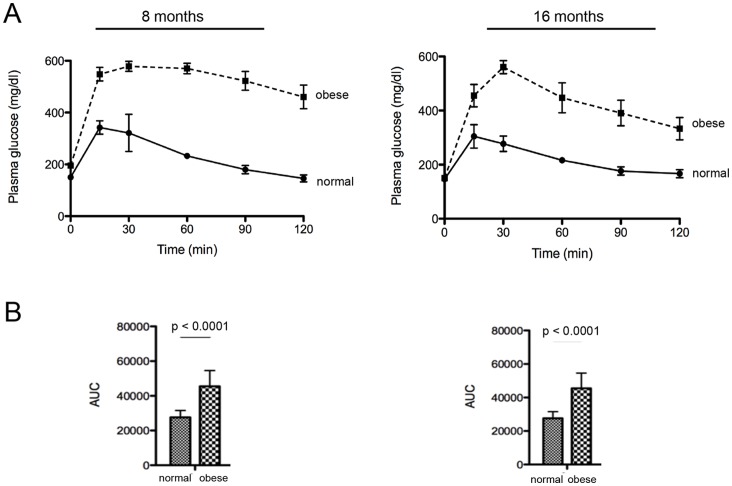
Metabolic features of normal and obese mice. (A) Representative glucose tolerance curves at eight and 16 months of normal and obese mice. (B) The area under the curve (AUC) of glucose tolerance test was calculated for each animal using the trapezoidal rule. n = 10. Mean ± SEM, p<0.0001 vs. normal mice (Student test).

Compared to animals fed the regular diet, animals fed with the high-fat diet for eight months presented overweight, hyperglycaemia, hyperinsulinemia, hypercholesterolemia (Table 2), and insulin resistance (Figure 1). In the obese mice, these metabolic parameters remained altered up to the end of the study period.

**Table 2:** Biochemical Markers Related to Metabolic Syndrome.

Time (Months)	8		12		16	
Groups	Normal	Obese	Normal	Obese	Normal	Obese
**Body Weight (g)**	26±1	42±1*	35±2	52±3*	35±3	57±3*
**Glucose (mg/dl)**	119±3	151±7**	135±5	154±6*	134±6	158±5*
**Insulin (µg/l)**	0.7±0.1	2.4±0.2*	0.9±0.1	5.2±0.4*	0.8±0.1	4.8±0.3*
**Triglycerides (mg/dl)**	104±4	113±5	119±15	125±9	82±9	90±9
**Cholesterol (mg/dl)**	108±7	220±6**	112±9	197±24**	156±6	271±25*

Results are expressed in mean ± SEM. Student test vs. normal mice,

* p<0.05,

** p<0.01, n = 10.

### High-fat Diet-induced Obese Mice show Normal Blood Pressure Parameters

**Figure 2:**
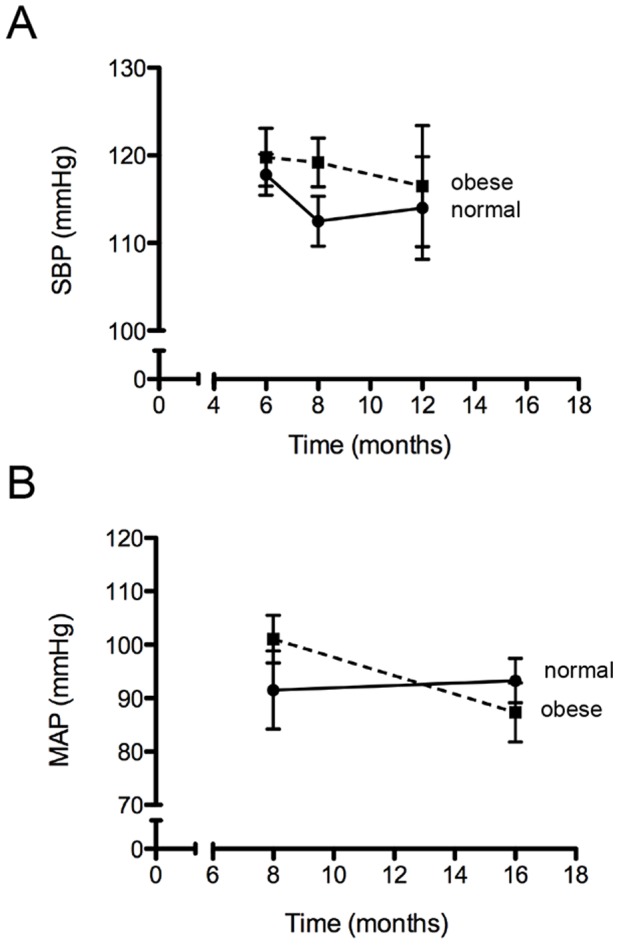
Blood pressure parameters of normal and obese mice. (A) Systolic blood pressure (SBP) was determined with sphygmomanometer (non-invasive determination) up to 12 months. (B) Mean arterial pressure was determined with cardiac catheterization (invasive determination) at eight and 16 months. n = 5–6. Mean ± SEM.

Irrespective of the methodology used to measure blood pressure (non-invasive: SBP or invasive: MAP), no statistically significant differences were observed between normal and obese mice at the time point studied (Figure 2).

### High-fat Diet-induced Obese Mice Present Reduced Aortic Vasoconstriction

**Figure 3:**
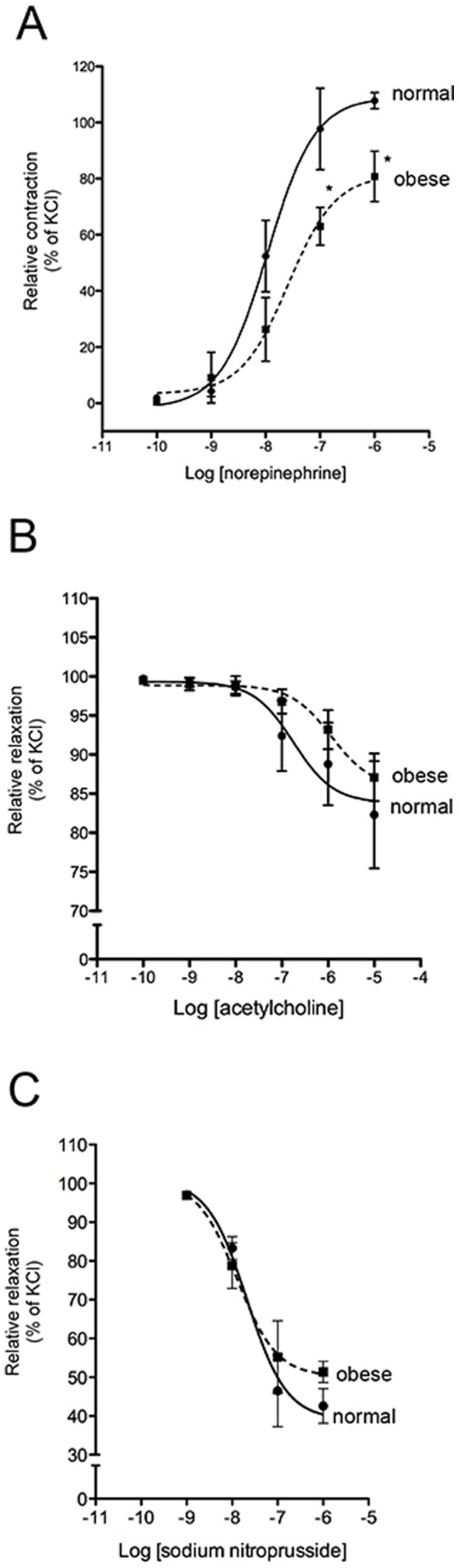
Vascular reactivity of normal and obese mice. The aortic ring of normal and obese mice was exposed to different vasoactive agents at 16 months. (A) Vasoconstriction by norepinephrine. (B) Vasorelaxation by acetylcoline. (C) Vasorelaxation by sodium nitroprusside. n = 4. Mean ± SEM, p<0.05 vs. normal mice (Two-way ANOVA test).

At month eight, no differences were detected in the vascular reactivity of normal and obese mice (data not shown). At month 16, vascular constriction in response to norepinephrine was reduced in obese mice compared to normal mice (Figure 3a). The EC_50_ values of norepinephrine were significantly different (1.1×10^−2^ µM *vs*. 2.6×10^−2^ µM, p<0.03). Vascular relaxation in response to acetylcholine was lower in obese mice compared with normal mice, but not statistically significant (Figure 3b). Vascular relaxation in response to sodium nitroprusside was similar in both experimental groups (Figure 3c).

### High-fat Diet-induced Obese Mice Display Normal Cardiac Function under Basal Conditions but Dysfunction is Unmasked under Stress Conditions

**Figure 4:**
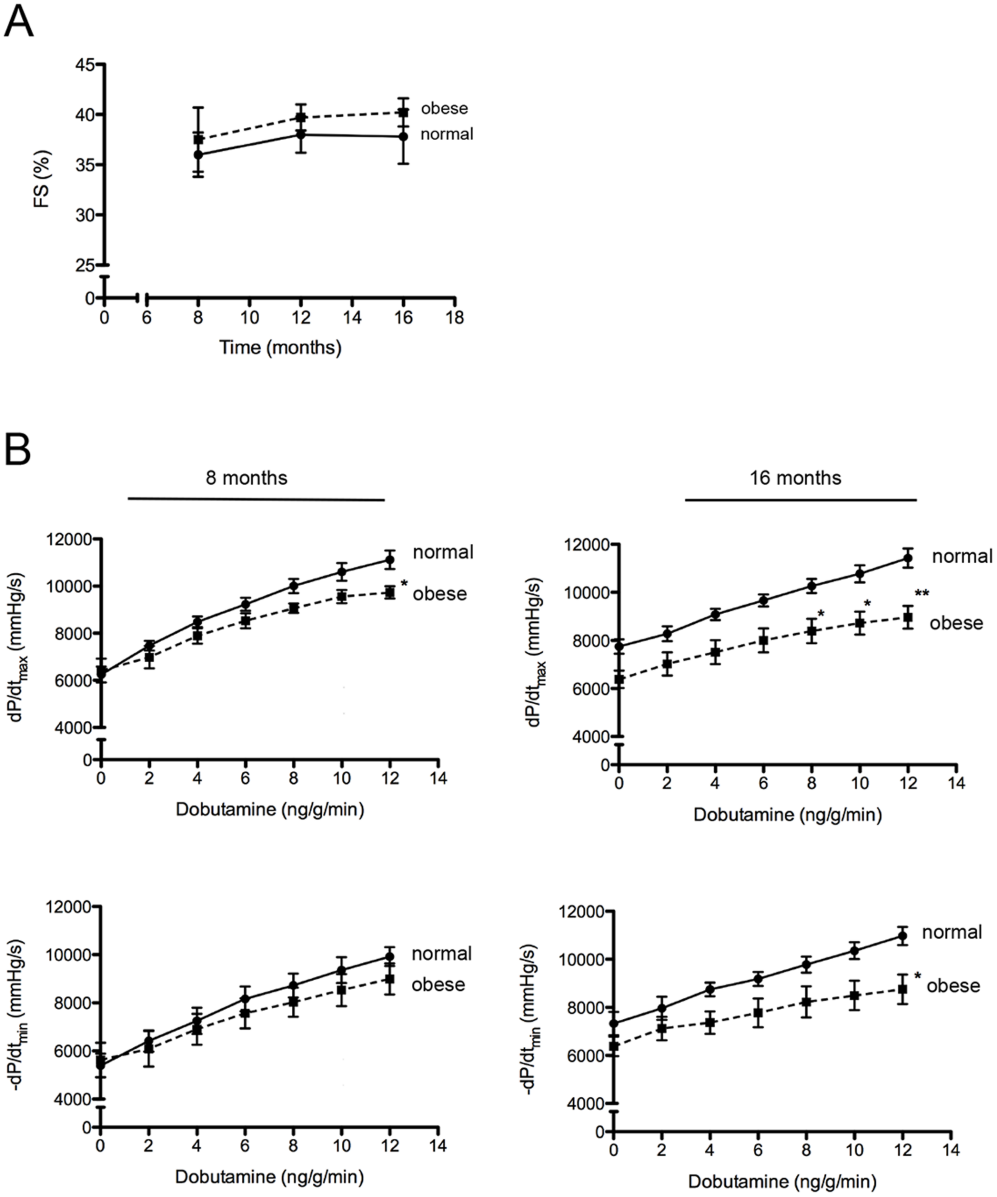
Cardiac function under basal and stress conditions of normal and obese mice. (A) Fractional shortening (FS%) was monitored by echocardiography under basal conditions at eight, 12 and 16 months. (B) Cardiac catheterization under basal and stress conditions was performed in order to obtained maximal positive pressure development (dP/dt_max_), maximal negative pressure development (dP/dt_min_). n = 5–7. Mean ± SEM, *: p<0.05, **: p<0.01 vs. normal mice (Two-way ANOVA test).

Under basal conditions, no significant changes were observed in FS, maximal positive pressure development (dP/dt_max_) and maximal negative pressure development (dP/dt_min_), between normal and obese mice (Figure 4). Nevertheless, when cardiac function was evaluated under pharmacologic stress, obese mice showed a reduced contractile response (lower dP/dt_max_), which worsened from eight to 16 months of feeding the high-fat diet (Figure 4b). At month 16, cardiac relaxation response (dP/dt_min_) was also impaired in obese mice compared with normal mice.

### High-fat Diet-induced Obese Mice Exhibit Cardiac Remodelling

**Figure 5:**
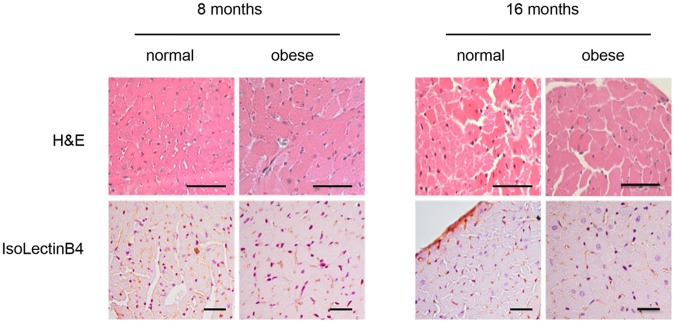
Light microscopy features of cardiac structure of normal and obese mice. Transversal heart sections were stained with haematoxylin/eosin (H&E) in order to measure the mean cross-sectional area of cardiomyocyte. Capillary density was determined using IsoLectinB4 that specifically detects endothelial cells. Images are representative of six animals per group. Scale bars = 50 um.

**Figure 6:**
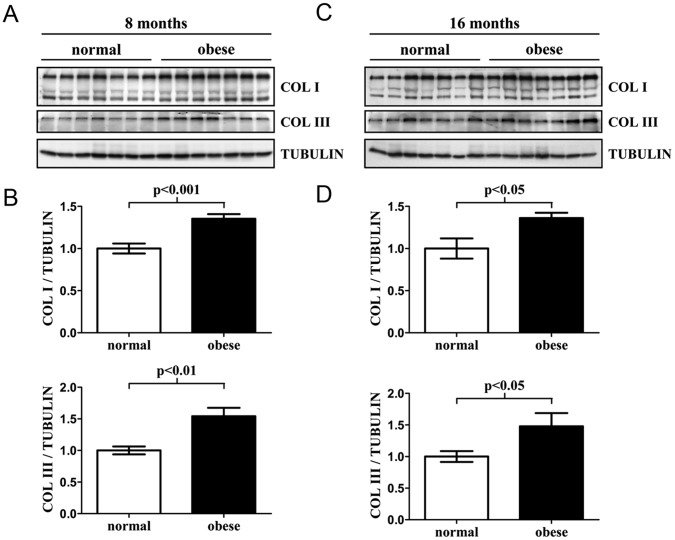
Collagen type I and collagen type III content in myocardium of normal and obese mice. (A and C) Representative immunoblots at eight and 16 months. Tubulin was detected as a loading control. (B and D) Densitometric measurements (arbitrary units). n = 7–8. p<0.05 vs. normal mice (Student test). COL I: collagen type I, COL III: collagen type III.

After eight months of high-fat diet feeding, a significant increase in heart size was observed in obese mice (Table 3). These animals also exhibited a thickening of both the intraventricular septum and left ventricular wall, with no change in its internal diameter. Thus, obese mice exhibited cardiac remodelling. As alterations are evidenced in the absence of ventricular dilatation, obese mice hearts underwent a concentric hypertrophy. Accordingly, cardiac fibers were thickened in obese mice compared with normal mice (Figure 5 and Table 3). The number of capillaries per cardiomyocyte remained unaltered in obese mice. Cardiac remodelling developed by obese mice was also confirmed by the overexpression of collagen types I and III, at both mRNA and protein levels (Table 4 and Figure 6). Furthermore, the expression of genes related to heart contractility (*alpha*-*MHC*, *beta*-*MHC* and *SERCA2A*) was unaltered by high-fat diet feeding, except at month 16, when *beta*-*MHC* was increased (2-fold) in obese mice compared with normal mice (Table 4).

**Table 3:** Cardiac Structure.

Time (Months)	8			12				16		
Groups	Normal		Obese	Normal		Obese		Normal	Obese	
**Heart Weight (mg)**	134±3	175±7*			141±5	209±9*	158±8			190±7*
**Heart Weight/** **Tibia Length(mg/mm)**	7.3±0.2	9.5±0.4*			7.6±0.3	11.3±0.5*	8.4±0.3			10.4±0.4*
**IVSd (mm)**	0.63±0.06	0.80±0.06*			0.61±0.05	0.84±0.04*	0.58±0.05			0.81±0.07*
**LVPWd (mm)**	0.63±0.07	0.77±0.14*			0.67±0.05	0.85±0.05*	0.65±0.04			0.80±0.10*
**LVIDd (mm)**	4.02±0.19	3.95±0.29			4.09±0.16	4.22±0.12	3.98±0.10			4.11±0.20
**A [cmy] (µm^2^)**	377±40	677±81*			397±34	672±41*	513±46			786±54*
**N°Capillaries per Cardiomyocyte**	2.9±0.2	3±0.1			3.5±0.1	3.6±0.2	3.6±0.2			3.7±0.2

Results are expressed as mean ± SEM. Two-way ANOVA test vs. normal mice.

* p<0.05, n = 6–8.

A[cmy]: mean cross-sectional area of cardiomyocytes, IVSd: interventricular septum, LVIDd: left ventricular internal diameter, LVPWd: left ventricular posterior wall at end diastolic measurements.

**Table 4:** Cardiac Gene Expression.

Time (Months)	8		16	
Groups	Normal	Obese	Normal	Obese
*GLUT1*	1,00±0,14	0,51±0,10*	1,00±0,27	1,01±0,36
*GLUT4*	1,00±0,16	0,51±0,06*	1,00±0,11	0,73±0,08
*PPAR-α*	1,00±0,19	0,96±0,16	1,00±0,21	1,63±0,36
*PDK4*	1,00±0,28	0,79±0,21	1,00±0,22	4,88±1,24#
*UCP3*	1,00±0,15	1,34±0,14	1,00±0,63	2,47±0,47*
*α-MHC*	1,00±0,20	0,78±0,19	1,00±0,33	1,58±0,35
*β-MHC*	1,00±0,12	0,97±0,12	1,00±0,16	2,06±0,26**
*SERCA2A*	1,00±0,11	0,97±0,19	1,00±0,22	1,38±0,21
*COL I*	1,00±0,17	1,66±0,20*	1,00±0,12	1,48±0,18*
*COL III*	1,00±0,15	1,85±0,76*	1,00±0,05	1,80±0,20*

*COL I* : collagen type I, *COL III*: collagen type III, *α-MHC*: alpha-myosin heavy chain, *β-MHC*: beta-myosin heavy chain, *SERCA2A*: sarco/endoplasmatic reticulum calcium ATPase, *GLUT1:* glucose transporter 1, *GLUT4:* glucose transporter 4, *PPAR-α*: peroxisome proliferator-activated alpha receptor, *PDK4:* pyruvate dehydrogenase kinase isozyme 4, *UCP3:* uncoupling protein 3. Results are expressed in mean ± SEM. Student test vs. normal mice.

* p<0.05,

** p<0.01, n = 7–8. Mann-Whitney test vs. normal mice.

# p<0.05, n = 8.

### High-fat Diet-induced Obese Mice Develop Cardiac Metabolic Compensation

At month eight, the mRNA levels of carbohydrate metabolic markers (*GLUT1* and *GLUT4*) appeared diminished in obese mice compared with normal mice (Table 4). At month 16, the gene expression of the lipid metabolic marker (*PPAR-alpha?* tended to increase in obese mice. The same was observed for *PDK4* and *UCP3*, genes that exacerbate cardiomyopathy.

## Discussion

In this work we evaluated the effect of feeding a high-fat diet for up to 16 months on the male C57BL/6 mice cardiovascular system. In obese mice, metabolic syndrome signs (hyperglycaemia, insulin resistance, hyperinsulinemia and hypercholesterolemia) gradually developed, and remained stable from month eight up to the end of the study. According to previous reports, the carbohydrates present on the high-fat diet (maltodextrin and a low-sucrose concentration) promote the onset, and lipids sustain the insulin resistance [28], [29].

Arterial pressure remained unchanged in mice throughout the entire study. At month eight, in obese mice a minor hypertension was observed but it did not significantly progress. To better understand this pressure decrease, arterial reactivity was studied. At month 16, the response to vasoconstriction agent (norepinephrine) was reduced in animals fed with the high-fat diet compared with normal mice. Previously, it has been shown that senescence-accelerated prone (SAMP8) mice fed with high-fat diet underwent a reduction in artery contraction stimulated by phenylephrine, and postulated it as an adaptive mechanism of aged mice to obesity [30]. The impairment in vasoconstriction secondary to the intake of a high-fat diet might be attributed to alterations in the synthesis of nitric oxide (NO). While in physiological conditions NO is mainly synthesized by endothelial nitric oxide synthase (eNOS), in pathological conditions inducible (iNOS) or neuronal (nNOS) are involved [31]. On another hand, in obese mice the vasorelaxation response to acetylcholine tends to reduce but was not statistically significant [32]. Thus, the arterial pressure pattern seen in long-term high-fat diet-induced obese mice might be explained by a deficiency in vasoconstriction resistance and the activation of the sympathetic nervous system secondary to insulin-resistance [33]. In support of this, it has been reported that obesity reduced the vascular adrenergic reactivity by a sympatho-mediated leptin-specific mechanism [34]. A high-fat diet feeding induces hyperleptinemia both in mice and humans [28], [35], [36]. Furthermore, genetically-modified mice models of type 2 diabetes (*db/db* or *ob/ob*) present both leptin metabolism disorder and hypertension [34], [37].

Regarding to cardiac performance, it has been shown that beta-adrenergic stimulation is compromised in cardiomyocyte that accumulates lipids [22], [38]. Nevertheless, in obese mice we did not see consistent decrease in heart rate when compared with normal mice (data not shown). Cardiac stress test is often used to unmask subclinical diseases that at baseline conditions are undetectable [39]. At month 8 and 16, compared with normal mice, in obese mice we observed no change under basal condition, but a reduction of heart inotropic response to dobutamine. This is in agreement to previously report data showing in the *db/db* mice an impaired cardiac functional reserve upon dobutamine infusion [22].

In obese mice the results show concentric cardiac hypertrophy and cardiomyocyte hypertrophy, but no change in capillary density. The former is in agreement with findings from the Framingham Heart Study, which showed a marked association between insulin resistance secondary to obesity and increased ventricular size [40]. A similar cardiac phenotype was reported when using diets that promote diabetes, but not obesity [41], [42]. Hyperglycemia increases myocardial production of angiotensin II and the renin-angiotensin system was associated with hypertrophy of cardiomyocyte and increase formation of glucose-derived advanced glycation end products, which contribute to myocardial stiffness [43]–[45]. On another hand, overexpression of collagen types I and III was associated with diastolic dysfunction because of their negative effects on the elastic properties of the heart wall [46].

Insulin resistance produces a change in the energetic metabolism in the heart, decreasing glucose uptake and increasing lipid uptake [38]. As a result, cardiac lipid accumulation promotes a change in several genes that regulate glucose-lipid utilization in order to obtain energy [47]. After 16 months of high-fat diet feeding, an overexpression of *PPAR-alpha* and *PDK4* was found. Those factors promote lipid utilization as an energy substrate for mitochondrial oxidation. Furthermore, it has been shown that restrained expression of *PPAR-alpha* results in a cardiac phenotype similar to the one secondary to diabetes [48]. The overexpression of *UCP3* has been associated with a mitochondrial uncoupling process that reduces cardiac efficiency [49], [50]. The overexpression of *beta-MHC* is an adaptation mechanism to preserve energy and reduce contractile function when glycolytic activity decreases and lipid oxidation increases [51], [52]. Also, *beta-MHC* overexpression is considered as a marker of pathological cardiac hypertrophy [53]. Together, these data show that the hearts of obese mice underwent an adaptive response to metabolic change. This adaptation is sufficient to maintain cardiac function under basal condition but not under stress.

Hence, we show that male C57BL/6 mice fed with a high-fat diet for 16 months (17 months old) presented overweight, hyperglycaemic, insulin resistant, hyperinsulinemic, hypercholesterolemic, normal arterial pressure with altered vascular reactivity (vasoconstriction), and cardiac contractility reserve reduction, heart mass increase, cardiomyocyte hypertrophy, cardiac fibrosis, and heart metabolic compensations. By contrast, mice fed with regular diet remained healthy throughout the study. Since, diabetic cardiomyopathy has been defined as a ventricular dysfunction with absence of hypertension and coronary artery disease developed in diabetic patients [54], [55], here we describe an animal model that recapitulates not only the etiology but also the course and the outcome of the early phases of human diabetic cardiomyopathy [11]. The main practical constrains of the model here presented are: i) 15% of the mice gain weight with a tardy kinetic or do not become obese [56], ii) 10% of the mice develop ulcerative dermatitis and must be withdraw from the study, iii) significant space and high quality standard procedures must be offered in the animal facility in order to keep a major number of animals for more than one year. This animal model of early phases of diabetic cardiomyopathy might be a useful tool to perform pre-clinical testing of new diagnostic, prevention and curative strategies for a disease that has gained more relevance during the last decade [57], [58].
